# Supplementary material for: Pan-Cancer Analysis Reveals Disulfidoptosis-Associated Genes as Promising Immunotherapeutic Targets: Insights Gained from Bulk Omics and Single-Cell Sequencing Validation
Source: Biomedicines. 2024 Jan 24;12(2):267. doi: 10.3390/biomedicines12020267 (PMC10887130; doi:10.3390/biomedicines12020267)
Supplement: Supplementary file 1 [file biomedicines-12-00267-s001.zip › biomedicines-2683145-supplementary.pdf]

biomedicines

MDPI

Article

Supplementary Materials: Pan-Cancer Analysis Reveals Disulfidoptosis-Associated Genes as Promising Immunotherapeutic Targets: Insights Gained from Bulk Omics and Single-Cell Sequencing Validation

Borui Xu, Minghao Li, Nuoqing Weng, Chuzhou Zhou, Yinghui Chen, Jinhuan Wei and Liangmin Fu

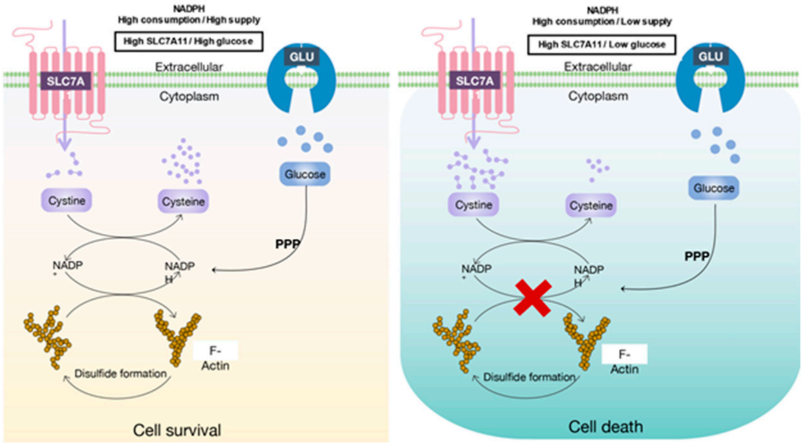

Supplementary Figure S1. Schematic Diagram of Disulfidoptosis Mechanism. SLC7A11 imports extracellular cystine in for exchange for intracellular glutamate., whereas SLC3A2 anchors SLC7A11 to the plasma membrane. Within the cell, cystine is reduced to cysteine, and γ-GCS catalyzes the synthesis of γ-glutamylcysteine from glutamate and cysteine. Then γ-glutamylcysteine and glycine are converted into glutathione by GS. Disulfidptosis is a new cell death, which mechanism is in SLC7A11high cells under glucose starvation, high cystine uptake coupled with the shortage of NADPH supply leads to NADPH depletion, aberrant disulfide bonding in actin cytoskeleton proteins, actin network collapse, and subsequent cell death. γ-GCS: γ-glutamylcysteine synthetase, GS: glutathione synthetase.

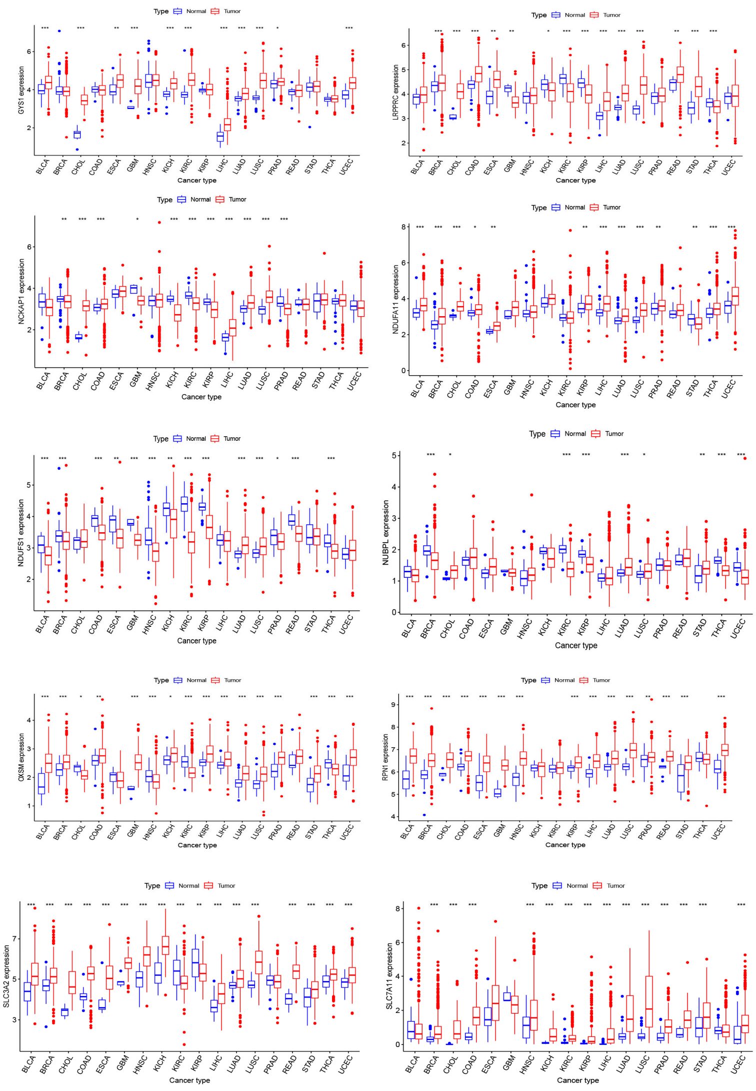

Supplementary Figure S2. Prognostic Value of disulfidoptosis-related genes in pancancer. Kaplan-Meier (KM) survival curve of other disulfidoptosis-related genes, including GYS1, NDUFS1, OXSM, LRPPRC, NDUFA11, NUBPL, NCKAP1, RPN1 and SLC3A2.

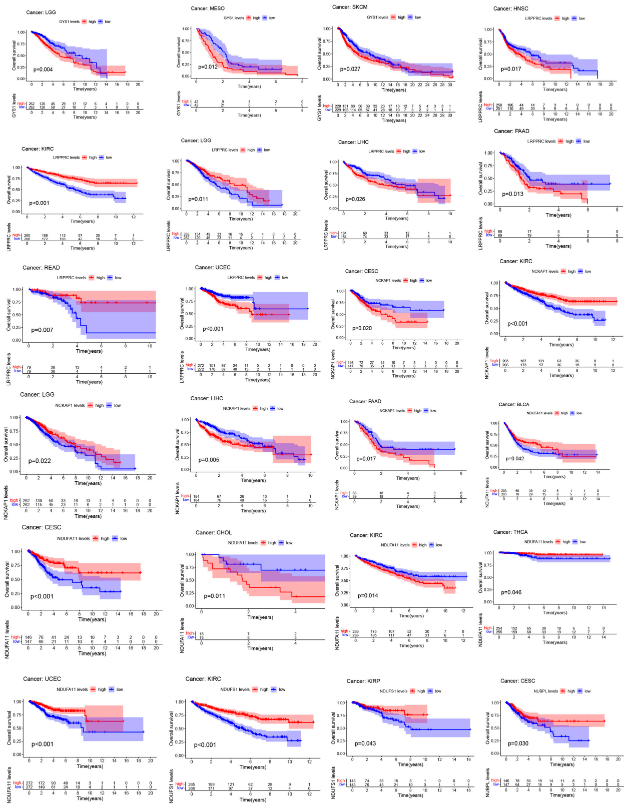

Supplementary Figure S3. The expression of disulfidoptosis-related genes in pancancers were analyzed in TCGA database. *P < 0.05; **P < 0.01; ***P < 0.001.

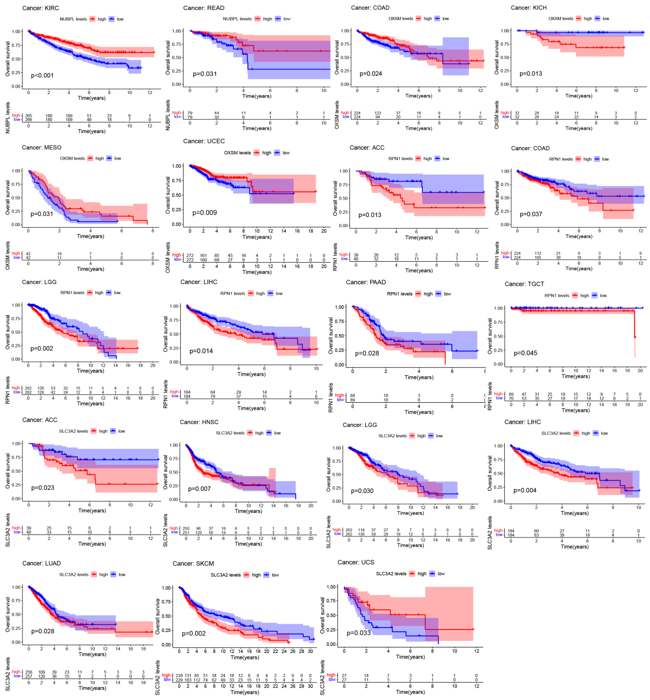

Supplementary Figure S4. The expression of disulfidoptosis-related genes in pancancers were analyzed in TCGA database. *P < 0.05; **P < 0.01; ***P < 0.001.

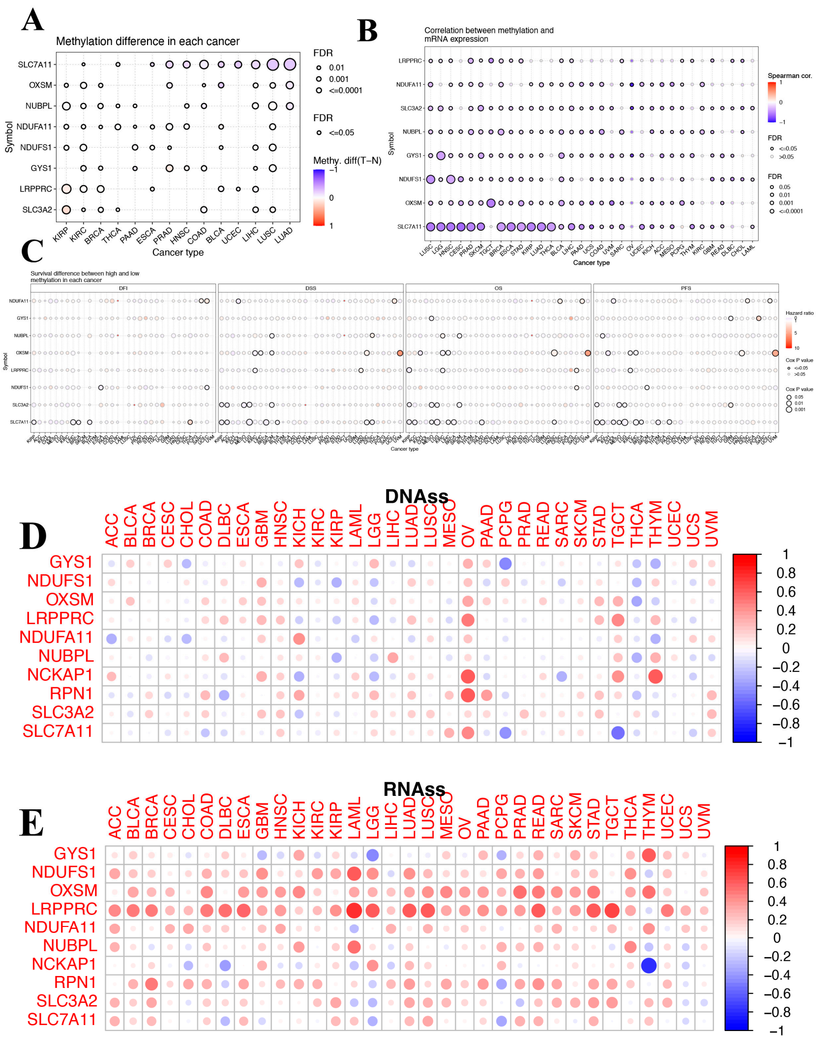

Supplementary Figure S5. Methylation and the stemness of tumors of disulfidoptosis-related genes in cancers. (A) Scatter plots of methylation different in pancancer. (B) Scatter plots of correlations between disulfidoptosis-related genes methylation and mRNA expression. (C) Prognostic value of disulfidoptosis-related prognostic model genes DNA methylation in HCC. (D,E) Relationships between expression of disulfidoptosis-related prognostic model genes and the stemness of tumors, including DNAss (D) and RNAss (E). Size of the circles indicates statistical significance (FDR) red: high level of immune cell infiltration; blue: low level of immune cell; Cor, correlation values; FDR, False-discovery rate. The figures A,B,C are derived from the GSCA database.

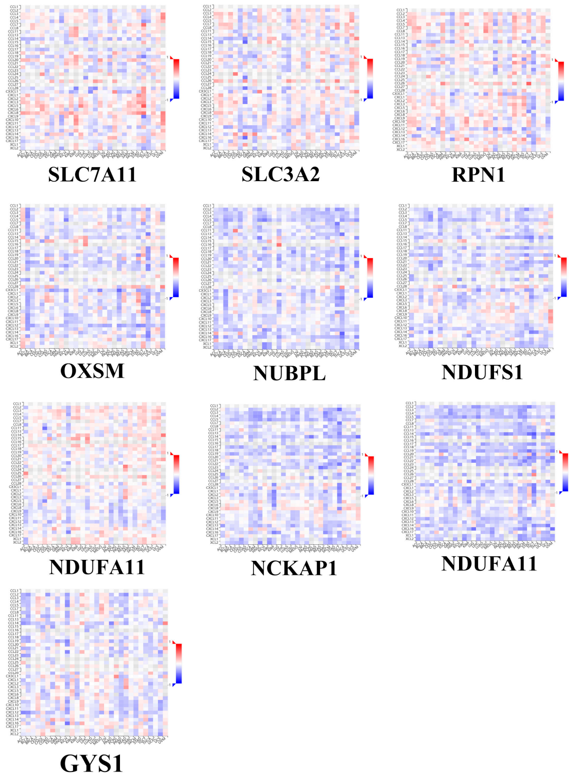

Supplementary Figure S6. The correlation between disulfidoptosis-related genes expression and chemokine-related genes. Red color means positive correlation, blue color means negative correlation.

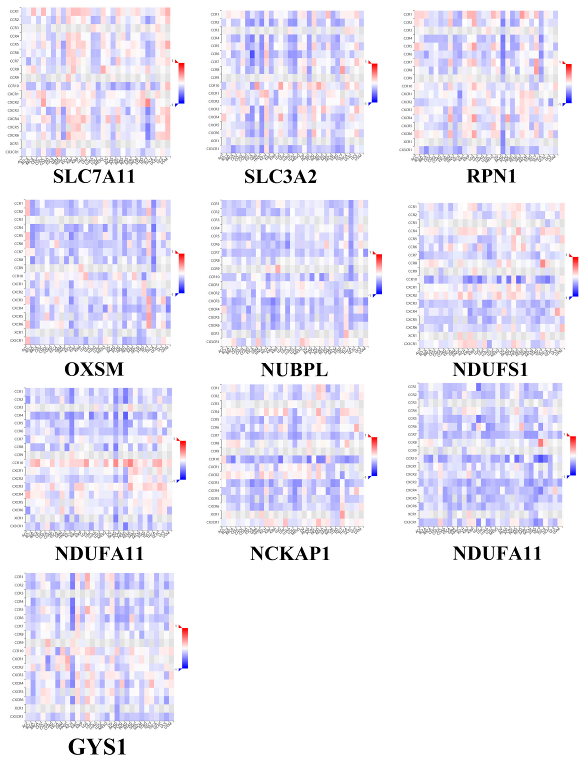

Supplementary Figure S7. The correlation between disulfidoptosis-related genes expression and chemokine receptor-related genes. Red color means positive correlation, blue color means negative correlation.

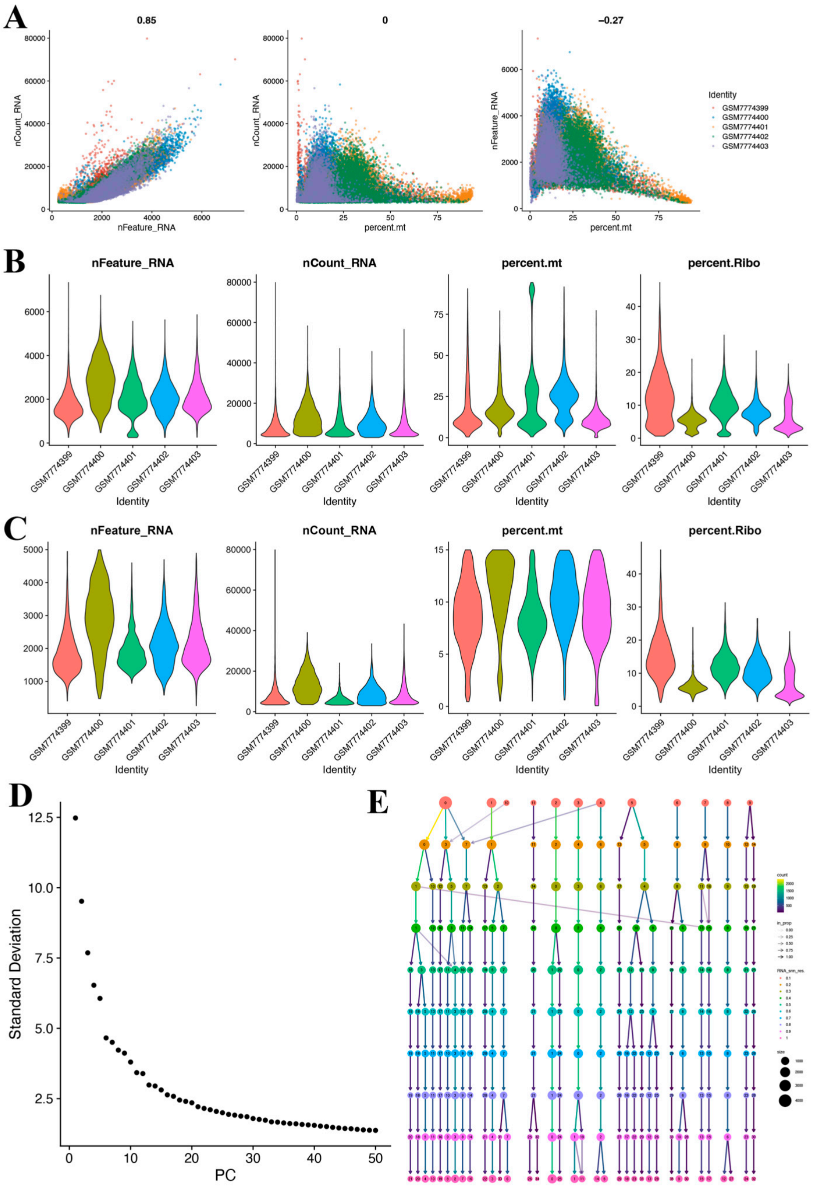

Supplementary Figure S8. Single-Cell Sequencing Analysis of HCC Specimens. (A) Correlational analysis encompassing nFeature vs. nCount, percent.mt vs. nCount, and percent.mt vs. nFeature. (B) Violin plots depicting the RNA characteristic count (nFeature RNA) and the absolute UMI count (nCount RNA) prior to cell quality control measures. (C) Violin plots showcasing the RNA characteristic count (nFeature RNA) and the absolute UMI count (nCount RNA) post quality control filtering. (D) Principal Component Analysis (PCA) executed on the single-cell RNA sequencing data. (E) Analytical clustering tree with resolution parameter set to 0.7.

Supplementary Table S1. Abbreviations of cancers in TCGA.

Abbreviation	Detail
ACC	Adrenocortical Cancer
BLCA	Bladder Cancer
BRCA	Breast Cancer
CESC	Cervical Cancer
CHOL	Bile Duct Cancer
COAD	Colon Cancer
COADREAD	Colon and Rectal Cancer
DLBC	Large B-cell Lymphoma
ESCA	Esophageal Cancer
FPPP	FFPE Pilot Phase II
GBM	Glioblastoma

GBMLGG	lower grade glioma and glioblastoma
HNSC	Head and Neck Cancer
KICH	Kidney Chromophobe
KIRC	Kidney Clear Cell Carcinoma
KIRP	Kidney Papillary Cell Carcinoma
LAML	Acute Myeloid Leukemia
LGG	Lower Grade Glioma
LIHC	Liver Cancer
LUAD	Lung Adenocarcinoma
LUNG	Lung Cancer
LUSC	Lung Squamous Cell Carcinoma
MESO	Mesothelioma
OV	Ovarian Cancer
PAAD	Pancreatic Cancer
PANCAN	Pan-Cancer
PCPG	Pheochromocytoma & Paraganglioma
PRAD	Prostate Cancer
READ	Rectal Cancer
SARC	Sarcoma
SKCM	Melanoma
STAD	Stomach Cancer
TGCT	Testicular Cancer
THCA	Thyroid Cancer
THYM	Thymoma
UCEC	Endometrioid Cancer
UCS	Uterine Carcinosarcoma
UVM	Ocular melanomas

Supplementary Table S2. Oligo sequences used in quantitative real-time PCR, inclduing Melting temperature (Tm) and Annealing temperature (Tm-5°C).

**Target**		Primer	Melting temperature (Tm)	Annealing temperature (Tm-5°C)
**GAPDH**	Forward	GAACGGGAAGCTCACTGG	63.0°C	58.0°C
	Reverse	GCCTGCTTCACCACCTTCT	65.3°C	60.3°C
SLC7A11	Forward	TCAGAAAGCCTGTTGTGTCCA	65.0°C	60.0°C
	Reverse	GACTTTCCTCTTCAGCTGCACTT	65.9°C	60.9°C

Supplementary Table S3. Two SLC7A11 siRNAs sequence.

**SLC7A11-siRNA1**	**CCUCUAUUCGGACCCAUUUTTAAAUGGGUCCGAAUAGAGGTT**
SLC7A11-siRNA2	GAAGUCUUUGGUCCAUUACTTGUAAUGGACCAAAGACUUCTT
